# Inter-individual variability in peripheral oxygen saturation and repeated sprint performance in hypoxia: an observational study of highly-trained subjects

**DOI:** 10.3389/fspor.2024.1452541

**Published:** 2024-08-08

**Authors:** Naoya Takei, Ryuji Muraki, Olivier Girard, Hideo Hatta

**Affiliations:** ^1^ Research Institute of Physical Fitness, Japan Women’s College of Physical Education, Tokyo, Japan; ^2^ Department of Sports Sciences, The University of Tokyo, Tokyo, Japan; ^3^ Department of Sports Science, Surugadai University, Saitama, Japan; ^4^ School of Human Sciences, Exercise and Sport Science, University of Western Australia, Perth, WA, Australia

**Keywords:** hypoxic training, simulated altitude, repeated sprint ability, oxygen saturation, individual variation

## Abstract

Individual variations in peripheral oxygen saturation (SpO_2_) during repeated sprints in hypoxia and their impact on exercise performance remain unclear despite fixed external hypoxic stimuli (inspired oxygen fraction: FiO_2_). This study examined SpO_2_ individual variations during repeated sprints in hypoxia and their impact on exercise performance. Thirteen highly-trained sprint runners performed 10 × 10-s cycle sprints with 30-s passive recoveries in normobaric hypoxia (FiO_2_: 0.150). Mean power output (MPO), post-sprint SpO_2_, and heart rate for each sprint were assessed. Sprint decrement score (S_dec_), evaluating fatigue development, was calculated using MPO variables. Participants were categorized into a high saturation group (HiSat, *n* = 7) or a low saturation group (LowSat, *n* = 6) based on their mean post-sprint SpO_2_ (measured 10–15 s after each sprint). Individual mean post-sprint SpO_2_ ranged from 91.6% to 82.2%. Mean post-sprint SpO_2_ was significantly higher (*P* < 0.001, *d* = 1.54) in HiSat (89.1% ± 1.5%) than LowSat (84.7% ± 1.6%). A significantly larger decrease in S_dec_ (*P* = 0.008, *d* = 1.68) occurred in LowSat (−22.3% ± 2.3%) compared to HiSat (−17.9% ± 2.5%). MPO (*P* = 0.342 *d* = 0.55) and heart rate (*P* = 0.225 *d* = 0.67) did not differ between groups. There was a significant correlation (*r* = 0.61; *P* = 0.028) between SpO_2_ and S_dec_. In highly-trained sprint runners, individual responses to hypoxia varied widely and significantly affected repeated sprint ability, with greater decreases in SpO_2_ associated with larger performance alterations (i.e., larger decrease in S_dec_).

## 1 Introduction

Repeated sprint training in hypoxia (RSH) is a well-established method to improve repeated sprint ability (1). A previous meta-analysis reported significant increases in peak (standard mean difference, SMD = 0.31) and mean (SMD = 0.46) power outputs during repeated sprint exercises in hypoxia compared to normoxic training (1). These performance improvements are notably attributed to physiological adaptations (e.g., improved blood perfusion glycolytic enzyme activity and pH regulation) via activation of hypoxia-inducible factor (HIF-1) (2, 3). To date, most RSH studies have used simulated altitudes of 2,000–3,000 m (inspired oxygen fraction or FiO_2_: 0.145–0.164), where adequate blood and tissue deoxygenation stimulate the HIF-1 pathway and promote physiological adaptations (1, 4, 5).

Despite the use of fixed “external” hypoxic stimuli (FiO_2_: 0.145–0.164) in previous studies, significant inter-individual variability persists in the “internal” hypoxic response (SpO_2_) (6, 7). Chapman et al. (6) reported substantial individual differences in SpO_2_ changes (ranging from 94.5% to 85.9%), noting that individuals with greater declines in SpO_2_ exhibited more pronounced decreases in 3,000 m running performance under moderate hypoxia (∼2,100 m above sea level) compared to normoxia. Therefore, an individual who exhibits a larger decrease in SpO_2_ may increase the internal physiological stimulus (e.g., activation of the HIF-1 pathway) while decreasing the external workload (e.g., reducing mechanical training load). Inter-individual variability in hypoxic response may affect both internal and external loads, influencing training effectiveness. In fact, inter-individual variability may yield heterogenous physiological adaptations (ranging from −5% decrease to +25% increase in total hemoglobin mass) and performance improvement (ranging from −1% reduction to +10% improvement in velocity at onset of blood lactate accumulation) after a 3-week altitude training (8). Therefore, although positive effects have been documented on average, inter-individual variability during hypoxic training may cause some individuals to be non- or low responders. Despite the established efficacy of RSH to increase performance, assessments of performance improvements have often been confined to group mean responses, assuming minimal interindividual variability in the hypoxic stimulus. Therefore, this observational study of highly-trained subjects aimed to test the hypothesis that significant individual acute variations exist in SpO_2_ changes during repeated sprints in hypoxia at a specified external hypoxic stimulus (FiO_2_: 0.150), and that athletes experiencing a larger physiological stimulus (i.e., greater SpO_2_ decrease) also demonstrate larger performance decrements across sprint repetitions.

## 2 Materials and methods

### 2.1 Participants

Thirteen “highly-trained” male sprint runners (Age: 21.3 ± 1.2 years; height: 1.72 ± 0.06 m; body weight: 63.9 ± 6.6 kg), categorized by established criteria (personal best records within ∼20% of world-record performance; Tier 3) (9), volunteered after providing written informed consent. They had been enrolled in a specific athletics training program for at least three years, which mainly included sprinting, plyometric exercises, and resistance training (∼150 min of training per day, 5 days per week). All participants were born and lived near sea level and had not been exposed to hypoxic environments in the preceding 6 months. The study adhered to the Declaration of Helsinki, and received approval from the Research Ethics Committee of the University of Tokyo (No. 891).

### 2.2 Experimental trials

This study was an observational examination of exercise performance under hypoxic conditions. All participants were familiar with cycling exercises, regularly performing “all-out” sprint cycling as part of their training regimen. After self-selected warm-up exercises (e.g., walking, jogging, static and dynamic stretching) in normoxia, participants performed 3 × 10-s sprint cycles with increased voluntary effort levels (60%, 80%, 100% of maximal efforts) in normobaric hypoxia. Then, participants performed a repeated sprint test consisting of 10 × 10-s “all-out” sprint efforts with 30-s passive recovery in normobaric hypoxia. The workload was set at 5.0% of each participants' body weight for all cycling exercises. Participants were instructed to avoid taking any ergogenic substances (e.g., supplements/energy drinks) for 24 h prior to the test and to avoid strenuous physical activity for 48 h prior. Testing took place at a consistent time of day (1–3 pm) to mitigate the influence of circadian rhythms, as different times of day can affect sprint performance (10).

### 2.3 Measures

The hypoxic chamber (FCC-5000S, Fuji Medical Science, Japan) provided a continuously regulated normobaric hypoxic environment (15% O_2_ and <1% CO_2_) through nitrogen dilution. Testing utilized an electrically braked cycle ergometer (PowerMax VⅢ, Konami, Japan), which enabled measurement of mean power output (MPO) for each sprint. The fatigue index (FI) and sprint decrement score (S_dec_) was calculated by the formula below (10).

(1) $$\mathrm{FI}(\text{\%})=100\times \frac{(S_{\mathrm{best}}\;-\;S_{\mathrm{worst}})\;}{S_{\mathrm{best}}}$$

(2) $$S_{\mathrm{dec}}(\text{\%})=\left\{\frac{\left(S_{1}+S_{2}+S_{3}+\ldots +S_{\mathrm{final}}\right)}{\left(S_{\mathrm{best}}\;\times \;\mathrm{number}\;\mathrm{of}\;\mathrm{sprints}\right)}-1\right\}\;\times 100$$

where *S* refers to sprint performance (i.e., MPO). The FI (Equation 1) assesses fatigue as the drop-off in performance from the best to worst sprints during repeated sprints. The S_dec_ (Equation 2) assesses fatigue by evaluating an individual's actual performance in relation to their “ideal performance” (i.e., where the best effort would be replicated in each sprint). Heart rate (Polar H10, Polar, Finland) and SpO_2_ (peripheral oxygen saturation) were measured after a 5-min seated rest under normoxia (normoxic baseline) and immediately (10–15 s) after each sprint. SpO_2_ was measured using a pulse oximeter (SAT-2200, Nihonkohden, Japan) on the fingertip of the index finger of the non-dominant hand. Participants were instructed not to grip the handle with their index finger. Participants were divided into two groups based on pooled mean post-sprint SpO_2_: those with SpO_2_ higher than the pooled mean value (87.1%) were assigned to the high saturation group (HiSat, *n* = 7), while those with lower SpO_2_ were placed in the low saturation group (LowSat, *n* = 6), allowing assessment of individual responses to hypoxia.

### 2.4 Statistical analysis

All values are expressed as mean ± SD. Differences were analyzed using paired *t*-tests or two-way repeated measures ANOVA [repetition (sprint 1, 2, 3 …, 10) × group (HiSat, LowSat)]. Tukey's multiple comparisons test was used for *post-hoc* pairwise comparisons to identify significant effects. Pearson's correlation analysis was performed to investigate relationships between variables. The magnitude of changes in the variables was expressed using standardized effect size (Cohen's *d*). Statistical significance was set at *P* < 0.05.

## 3 Results

The mean post-sprint SpO_2_ varied individually (87.1% ± 2.8%; ranging from 91.6% to 82.2%). SpO_2_ significantly decreased (*P* < 0.001) across repetitions, with a significant difference between groups (*P* < 0.001; Table 1). Significant effects of repetition (*P* < 0.001) and interaction (*P* < 0.001) were observed for MPO, while no significant effect of group was noted (*P* = 0.340; Table 1). Heart rate significantly increased (*P* < 0.001) across repetitions, independently of group (*P* = 0.173; Table 1).

**Table 1:** Time course changes in physiological and performance variables.

Variables		Sprint 1	Sprint 2	Sprint 3	Sprint 4	Sprint 5	Sprint 6	Sprint 7	Sprint 8	Sprint 9	Sprint 10	ΔSprint 1–10, %	*P* value		
													R	G	I
SpO_2_, %	HiSat	93.7 ± 1.7	92.7 ± 1.8	92.4 ± 1.6	91.0 ± 3.6	90.9 ± 2.6	87.9 ± 2.4^∼^	88.1 ± 3.1^∼^	85.7 ± 4.1^∼^	84.4 ± 4.3^∼^	84.3 ± 4.3^∼^	−10.1 ± 4.8	**<0**.**001**	**<0**.**001**	0.607
	LowSat	90.5 ± 2.7*	88.3 ± 2.9*	87.3 ± 4.1*	87.8 ± 1.0*	86.8 ± 3.1*	83.3 ± 2.7*^,^^∼^	81.5 ± 3.0*^,^^∼^	82.0 ± 4.4*^,^^∼^	80.3 ± 4.5*^,^^∼^	79.0 ± 3.7*^,^^∼^	−12.7 ± 3.8			
MPO, W/kg	HiSat	8.23 ± 0.50	7.30 ± 0.35^∼^	6.82 ± 0.53^∼^	6.85 ± 0.53^∼^	6.62 ± 0.53^∼^	6.56 ± 0.54^∼^	6.38 ± 0.44^∼^	6.39 ± 0.57^∼^	6.25 ± 0.67^∼^	6.25 ± 0.67^∼^	−25.0 ± 4.2	**<0**.**001**	0.340	**<0**.**001**
	LowSat	8.35 ± 0.71	7.42 ± 0.70^∼^	6.89 ± 0.59^∼^^,^^#^	6.45 ± 0.59^∼^	6.26 ± 0.51^∼^	6.15 ± 0.51^∼^	5.98 ± 0.44^∼^	5.82 ± 0.47^∼^	5.71 ± 0.36^∼^	5.86 ± 0.34^∼^	−29.7 ± 3.8			
Heart rate, bpm	HiSat	159.6 ± 13.3	168.7 ± 10.6	175.4 ± 11.3	177.9 ± 10.3	179.0 ± 10.4	177.6 ± 9.8^∼^	175.6 ± 7.5	176.7 ± 6.3	178.3 ± 8.8^∼^	180.1 ± 7.4^∼^	13.4 ± 7.8	**<0**.**001**	0.173	0.681
	LowSat	156.3 ± 10.9	166.5 ± 10.9	169.3 ± 8.30	172.0 ± 7.1^∼^	174.5 ± 4.9^∼^	171.2 ± 9.6	169.2 ± 6.2	174.8 ± 9.8^∼^	168.8 ± 10.9	173.3 ± 5.0	11.2 ± 6.9			

R, repetition; G, group; I, interaction; SpO2, peripheral oxygen saturation; HiSat, high saturation group; LowSat, low saturation group. All values are expressed as mean ± SD. ΔSprint1–10 is shown as percentage change between sprint 1 and sprint 10.

* Significantly different from HiSat (*P* < .05).

^∼^ Significantly different from sprint 1 (*P* < .05).

^#^ Significantly different from previous sprint (*P* < .05).

Bold *P* values indicate statistical significance.

**Figure 1:**
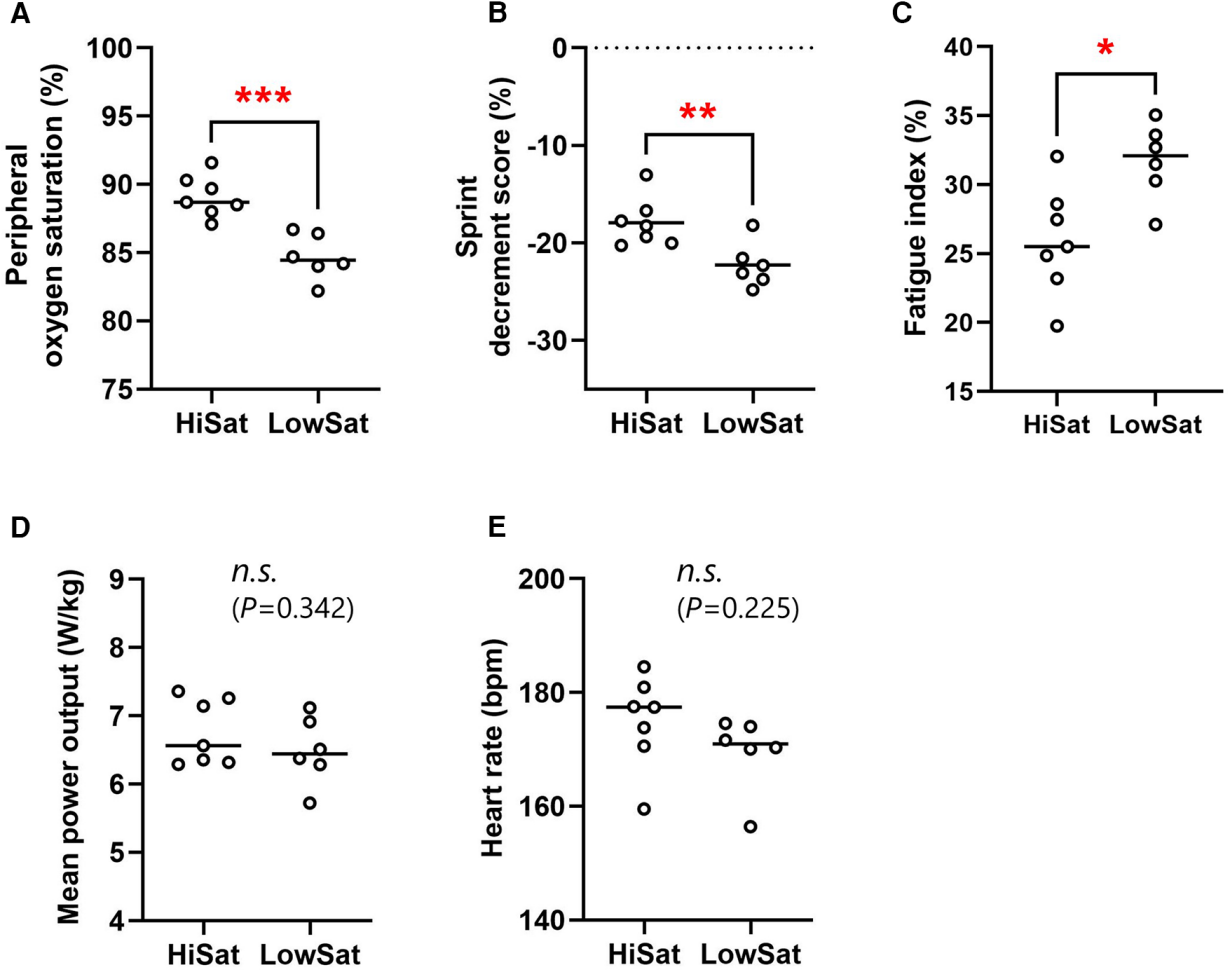
Peripheral oxygen saturation (**A**), sprint decrement score (**B**), fatigue index (**C**), mean power output (**D**), and heart rate (**E**) for high and low saturation groups (hiSat and lowSat, respectively). HiSat, high saturation group; LowSat, low saturation group. Peripheral oxygen saturation, mean power output and heart rate values were expressed as the mean value for all sprints. Circle markers indicate the individual measured values, and solid lines express the mean of each group. The *n.s.* indicates “not significantly different” between groups. *Significantly different between groups (*P* < 0.05) **Significantly different between groups (*P* < 0.01). ***Significantly different between groups (*P* < 0.001).

The normoxic baseline SpO_2_ did not differ between HiSat and LowSat (98.3 ± 0.8 vs. 98.2% ± 0.8%; *P* = 0.782). The mean post-sprint SpO_2_ was significantly higher (*P* < 0.001, *d *= 1.54) in HiSat than LowSat (89.1 ± 1.5 vs. 84.7% ± 1.6%, respectively; Figure 1A). FI (31.7 ± 2.8 vs. 25.9% ± 3.6%; *P* = 0.020 *d* = 1.53) and S_dec_ (−22.3 ± 2.3 vs. −17.9% ± 2.5%; *P* = 0.008 *d* = 1.68) were both worse in LowSat compared to HiSat (Figures 1B,C). MPO (6.76 ± 0.48 vs. 6.49 ± 0.49 W/kg; Figure 1D) and heart rate (174.9 ± 8.1 vs. 169.5 ± 6.7 bpm; Figure 1E) did not differ between HighSat and LowSat.

**Figure 2:**
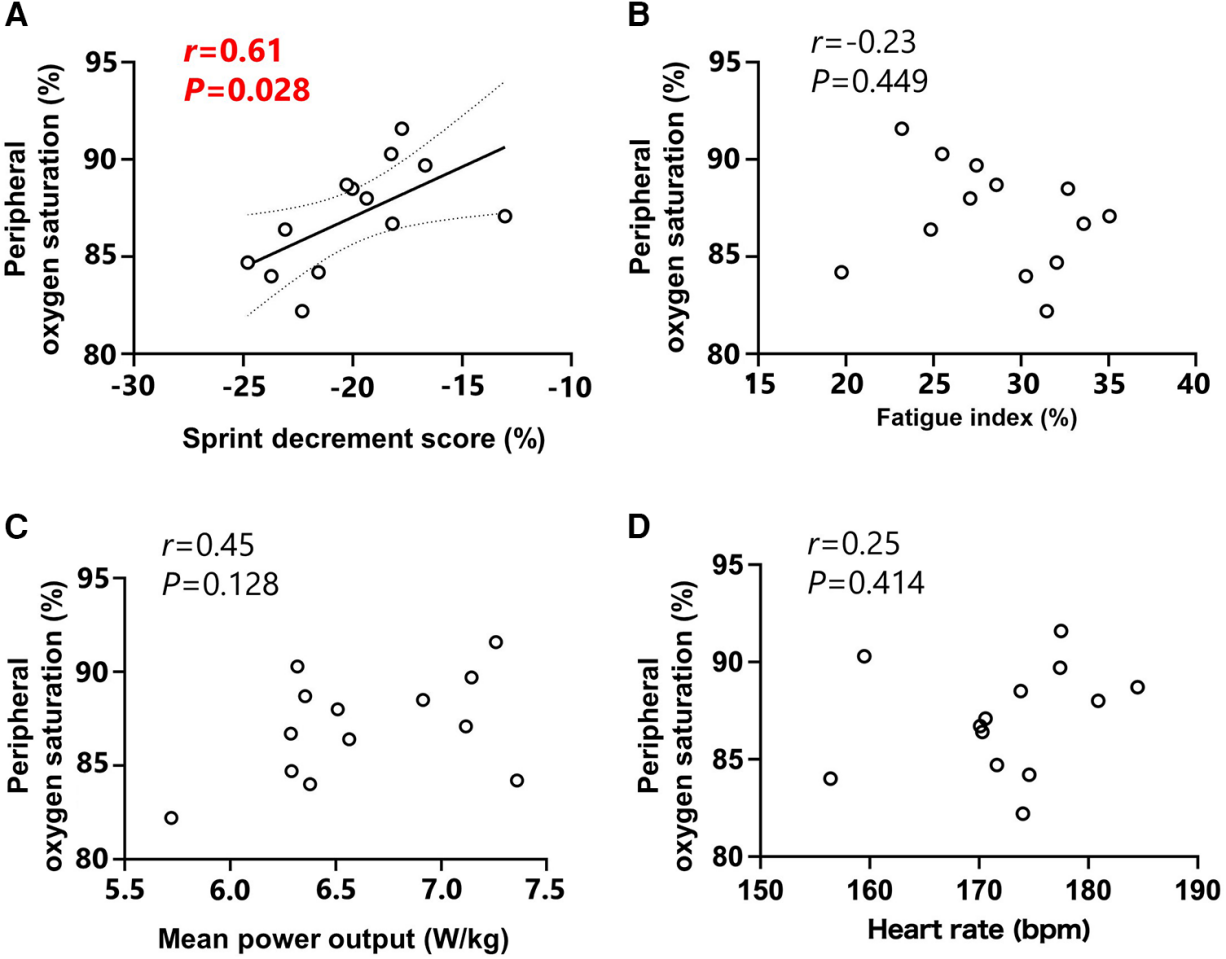
Relationships between peripheral oxygen saturation and sprint decrement score (**A**), fatigue index (**B**), mean power output (**C**) and heart rate (**D**) Peripheral oxygen saturation, mean power output and heart rate values were expressed as the mean value for all sprints. Circle markers indicate the individual measured values, and solid lines indicate approximate straight lines for all values. Dotted line expressed the area of 95% confidence interval.

The relationship between SpO_2_ and S_dec_ showed a significant correlation (*r* = 0.61, *P* = 0.028; Figure 2A), while no significant correlations were observed between SpO_2_ and FI (*r* = −0.23, *P* = 0.449; Figure 2B), MPO (*r* = 0.45, *P* = 0.128; Figure 2C) or heart rate (*r* = 0.25, *P* = 0.414; Figure 2D).

## 4 Discussion

Our study revealed significant individual variations in mean post-sprint SpO_2_ (ranging from 91.6% to 82.2%) under identical hypoxic conditions (FiO_2_: 0.150) in highly-trained sprint runners. These variations had a significant impact on repeated sprint ability, with participants in the LowSat group demonstrating deteriorated FI and S_dec_ compared to the HiSat group. Additionally, individual variations in mean post-sprint SpO_2_ significantly correlated with S_dec_, indicating that greater decreases in SpO_2_ were associated with greater decreases in performance, and *vice versa*. Although S_dec_ was significantly correlated with SpO_2_, there was no significant correlation observed between SpO_2_ and FI. This is likely because FI, which is based only on the best and worst sprints, can be is skewed by a single extremely fatiguing sprint and may not accurately reflect repeated sprint ability. Mean MPO also showed no difference between groups and no correlation with SpO_2_. In contrast, S_dec_, which evaluates fatigue across all sprints, is a better indicator of repeated sprint ability and demonstrates a significant relationship with SpO_2_. Previous research has reported that individual variability SpO_2_ affects 3,000-m running performance (6), suggesting that decreased SpO_2_ may reduce oxygen flow to working muscles, thus hampering endurance exercise performance. Our findings extend this understanding to repeated sprint exercise including ten “all out” sprint efforts (10 s) with short incomplete recovery (30 s). The influence of variations in blood oxygen saturation on repeated sprint ability may be attributed to decreased oxygen delivery to the skeletal muscles, which can lead to peripheral fatigue. Previous research employing neuromuscular assessments demonstrated that increasing severity of arterial hypoxemia leads to greater peripheral muscle fatigue during repeated treadmill sprints in severe hypoxia (FiO_2_: 0.13) compared to normoxia (FiO_2_: 0.21) and moderate hypoxia (FiO_2_: 0.17) (11). The mean post-sprint SpO_2_ in this previous study (83.7% ± 4.3%) was comparable to that of the LowSat group in our study (84.7% ± 1.7%) (11). Therefore, it is possible that pronounced decreases in SpO_2_ in LowSat vs. HiSat groups induced peripheral fatigue, resulting in deteriorated exercise performance (i.e., decreased S_dec_).

Although moderate hypoxia (FiO_2_: 0.150) was used in our study, large individual variations in response to hypoxia were observed, suggesting that some participants (LowSat) may face marked physiological stress (i.e., SpO_2 _< 85%), similar to those in severe hypoxic conditions reported in previous research (11). This indicates that while most previous RSH studies have used identical hypoxic conditions (FiO_2_: ∼0.145) for all their participants (1), the internal hypoxic response (SpO_2_) largely varies among individuals, and this variation may significantly affect training outcomes. Therefore, implementing personalized RSH programs that account for individual variations in response to hypoxia and adjust FiO_2_ levels accordingly is recommended. One potential explanation for the individual variation in internal hypoxic response (SpO_2_) under identical hypoxic environment could be individual differences in the ventilation response to hypoxic exposure (12). Previous studies have demonstrated that individuals experiencing exercise-induced arterial hypoxemia showed reduced VE/VO_2_ and end-tidal partial pressure of O_2_, along with increased end-tidal partial pressure of CO_2_, compared to individuals with non-exercise-induced arterial hypoxemia during exercise in both normoxia and hypoxia (12, 13). Additionally, Woorons et al. (13) reported a significant correlation (*r* = .55 to.73) between SpO_2_ and VE/VO_2_ across trained and untrained participants in different levels of hypoxia (FiO_2_: 0.187–0.117) (13). Therefore, individuals who fail to adequately increase VE in a hypoxic condition may experience pronounced hypoxemia. In this study, although not statistically significant, the MPO of the first three sprints was slightly higher in the LowSat group, with a larger performance decrement observed later. This may indicate that LowSat individuals had high anaerobic capacity but lower cardiovascular fitness.

One limitation of this study is the method of SpO_2_ measurement. We only measured baseline SpO_2_ under normoxia and post-sprint SpO_2_ under hypoxia. Therefore, it is unclear whether the observed inter-individual variability in SpO_2_ was induced by exercise or existed at rest in hypoxia. If variability exists at rest in hypoxia, it may serve as an indicator to predict exercise-induced internal hypoxic responses. This study also attempted to reduce the influence of peripheral vasoconstriction on SpO_2_ measurements by asking participants to hold the handle without using the index finger. However, the effect of peripheral vasoconstriction, which can overestimate peripheral deoxygenation, cannot be completely ruled out because blood flow to the fingertip was not monitored. Future studies measuring SpO_2_ at the forehead, which is unaffected by gripping the handle, could address this limitation.

In conclusion, despite being exposed to similar FiO_2_ levels, highly-trained sprint runners exhibit considerable variability in their responses to hypoxia, as indicated by their SpO_2_ readings. The magnitude of the hypoxic stimulus significantly influences repeated sprint ability, with individuals experiencing greater SpO_2_ decreases showing more pronounced impairments in performance, and *vice versa*.

## Data Availability

The raw data supporting the conclusions of this article will be made available by the authors, without undue reservation.
